# Carbon isotope ratios of endogenous steroids found in human serum—method development, validation, and reference population-derived thresholds

**DOI:** 10.1007/s00216-021-03439-9

**Published:** 2021-06-18

**Authors:** Thomas Piper, Hans Geyer, Eberhard Nieschlag, Lia Bally, Mario Thevis

**Affiliations:** 1 Center for Preventive Doping Research – Institute of Biochemistry, German Sport University Cologne, Am Sportpark Müngersdorf 6, 50933 Cologne, Germany; 2 Center of Reproductive Medicine and Andrology, University Hospital of Muenster, Domagkstraße 11, 48149 Münster, Germany; 3 Department of Diabetes, Endocrinology, Nutritional Medicine, and Metabolism, Inselspital, Bern University Hospital, University of Bern, 3010 Bern, Switzerland

**Keywords:** Serum, Steroids, Carbon isotope ratios, Doping controls, Multi-dimensional gas chromatography, IRMS

## Abstract

In order to detect the misuse of testosterone (T), urinary steroid concentrations and concentration ratios are quantified and monitored in a longitudinal manner to enable the identification of samples exhibiting atypical test results. These suspicious samples are then forwarded to isotope ratio mass spectrometry (IRMS)–based methods for confirmation. Especially concentration ratios like T over epitestosterone (E) or 5α-androstanediol over E proved to be valuable markers. Unfortunately, depending on the UGT2B17 genotype and/or the gender of the athlete, these markers may fail to provide evidence for T administrations when focusing exclusively on urine samples. In recent years, the potential of plasma steroids has been investigated and were found to be suitable to detect T administrations especially in female volunteers. A current drawback of this approach is the missing possibility to confirm that elevated steroid concentrations are solely derived from an administration of T and cannot be attributed to confounding factors. Therefore, an IRMS method for plasma steroids was developed and validated taking into account the comparably limited sample volume. As endogenous reference compounds, unconjugated cholesterol and dehydroepiandrosterone sulfate were found suitable, while androsterone and epiandrosterone (both sulfo-conjugated) were chosen as target analytes. The developed method is based on multi-dimensional gas chromatography coupled to IRMS in order to optimize the overall assay sensitivity. The approach was validated, and a reference population encompassing n = 65 males and females was investigated to calculate population-based thresholds. As proof-of-concept, samples from volunteers receiving T replacement therapies and excretion study samples were investigated.

**Graphical abstract:**

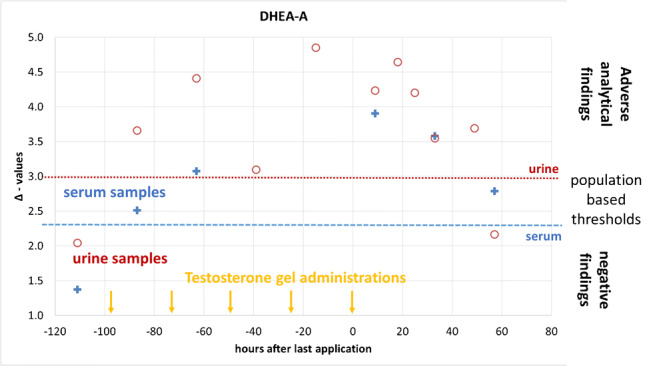

## Introduction

Since decades the detection of testosterone (T) or testosterone prohormone misuse in sports drug testing relies on the quantification of urinary steroids and ratios of concentrations like T divided by epitestosterone (E) [1]. Especially when applied in a longitudinal manner as exploited in the athlete biological passport, these markers are sensitive to the administration of T or T prohormones [2]. Unfortunately, numerous confounding factors can affect the T/E ratio in a similar way as T administrations [3, 4]. To the best of our knowledge, albeit possible, no adverse analytical finding has been established on the basis of the steroid profile alone since the steroidal module of the athlete biological passport (ABP) was launched, but samples suspicious for T administrations are forwarded to isotope ratio mass spectrometry (IRMS)–based methods in order to substantiate the exogenous origin of T or T metabolites [5].

In a recent publication, the limitations of the urinary steroid profile were demonstrated by data obtained on two female athletes, who did not respond to presumed T administrations by elevated concentration ratios in urine but only by significantly elevated T concentrations found in serum [6]. These results were corroborated by clinical trials encompassing low-dose administrations of T to female volunteers [7, 8]. In addition to T concentrations alone, methods for the determination of a comprehensive subset of serum steroids have been developed in the context of sports drug testing [9–11]. This will allow for a longitudinal steroid profile approach in parallel or as a complement to the current procedure based on urinary steroids. In contrast to the numerous confounding factors identified for urinary steroids, similar investigations for the serum steroid profile are still missing, with the exemption of data on the impact of ethanol consumption on serum T concentrations [12, 13]. In order to prevent time-consuming follow-up investigations on athletes found with abnormal serum steroid concentrations, an IRMS-based confirmation method to differentiate between T administrations and other possible confounding factors will be beneficial.

The determination of carbon isotope ratios (CIRs) to elucidate the source of urinary T and T metabolites has been established in sports drug testing more than 20 years ago [14]. The approach is based on the comparison of CIR of endogenous reference compounds (ERCs) not involved in T metabolism to the CIR of target compounds (TC) like T itself or its metabolites [5]. CIRs are expressed as δ^13^C-values against the international standard Vienna Pee Dee Belemnite (VPDB) following Eq. 1 and given in ‰ or mUr [15]:

1 $$ {\delta}^{13}{C}_{VPDB}=\frac{{\left(\frac{{}^{13}C}{{}_{12}C}\right)}_{SAMPLE}}{{\left(\frac{{}^{13}C}{{}_{12}C}\right)}_{VPDB}}-1 $$

Differences between an ERC and a TC are expressed as Δ-values:

2 $$ \Delta  \left[{\mbox{\fontencoding{U}\fontfamily{wasy}\selectfont\char104}} \right]={\delta}^{13}{C}_{ERC}-{\delta}^{13}{C}_{TC} $$

As the amount of serum available for analysis is limited and usually not more than 1 mL, those TCs showing the highest concentrations in serum had to be considered. Androsterone (A) and epiandrosterone (EpiA), both circulating as sulfated (S) phase II metabolites, were identified as possible targets. As ERCs dehydroepiandrosterone sulfate (DHEA-S) and unconjugated cholesterol (CHOL) were employed in the method. In order to avoid additional steps in sample preparation which might be accompanied by a loss of analyte and therefore diminish the recovery of the method, the use of liquid chromatography–based sample clean-up was circumvented. A multi-dimensional gas chromatography (MDGC)–based method prior to the IRMS was optimized to achieve signals of adequate peak purity, which is a prerequisite for IRMS determinations [16–19]. The developed method was validated taking into account current recommendations issued by the World Anti-Doping Agency (WADA) for urinary CIR [5], and a reference population was investigated to enable calculation of thresholds for different ERC-TC pairs and belonging Δ-values. As proof-of-concept, we investigated serum samples from subjects receiving T replacement therapy for male hypogonadism or participating in a T administration trial.

## Materials and methods

### Chemicals and steroids

Glacial acetic acid, sodium hydroxide (NaOH), methanol (MeOH), sulfuric acid (H_2_SO_4_), acetonitrile (ACN), *tert*-butyl methyl ether (TBME), ethyl acetate (EtOAc), and cyclohexane were purchased from Merck (Darmstadt, Germany). Pyridine was from Sigma-Aldrich (Steinheim, Germany) and acetic anhydride was a blend of reagents purchased from Sigma-Aldrich and Merck and vacuum distilled in-house before use. Solid phase extraction (SPE) cartridges (CHROMABOND C18 ec, 45 μm, 1 mL/100 mg) were purchased from Macherey & Nagel (Düren, Germany). Steroid reference materials A, EpiA, CHOL, DHEA, etiocholanolone (ETIO), and methyltestosterone (MeT) were supplied by Sigma-Aldrich and 5α-androstanediol (5a) and 5β-androstanediol (5b) by Steraloids (Newport, RI, USA). Certified steroid reference material for IRMS (USADA 33-1) was from Cornell University (Ithaca, NY, USA) employed to calibrate the CO_2_ tank gas (purity 4.5) provided by Linde (Pullach, Germany) [20]. Helium (purity 5.0 and 4.6), oxygen (purity 5.0), and hydrogen (purity 3.9) were also from Linde. Serum samples were collected in BD Vacutainer SST II 3.5-mL tubes (MedPlus, Radeberg, Germany).

### Sample preparation

To 1 mL of serum placed in a 15-mL sterile disposable centrifuge tube, 3 mL of ice-cold (−20 °C) ACN was added and the sample was vortexed thoroughly. After centrifugation (1800 rpm, 5 min), the supernatant was transferred to a new test tube and evaporated to dryness using compressed air at 50 °C. The dried residue was reconstituted with 2 mL of aqueous phosphate buffer (pH 7), 5 mL of TBME was added, and the sample was shaken for 5 min. After centrifugation, the organic layer containing unconjugated CHOL was transferred to a new test tube, evaporated to dryness, and kept for derivatization. After removing the remaining TBME layer, the aqueous residue was applied to a conditioned (2 mL of MeOH followed by 2 mL of water) SPE cartridge, washed with 2 mL of water, and eluted with 2 mL of MeOH. After evaporation to dryness, the acetic solvolysis was conducted by adding 2.5 mL of a mixture of EtOAc/MeOH (70/30, v/v) and 1 mL of a mixture containing 200 μL of H_2_SO_4_ in 100 mL EtOAc. The sample was incubated at 50 °C for 60 min and cooled to room temperature, and 0.5 mL of methanolic NaOH (1 M) was added before evaporation to dryness. The residue was reconstituted with 2 mL of water and extracted with TBME as described above. The organic layer containing formerly sulfated DHEA, EpiA, and A was transferred to a new test tube and evaporated. All samples were derivatized by adding 75 μL of pyridine and 75 μL of acetic anhydride and kept at 70 °C for 45 min. Afterwards, the samples were evaporated and reconstituted in 200 μL of cyclohexane and transferred to auto sampler vials with screw caps. As no method for the quantification of the serum steroids of interest was available at our laboratory, all samples were subjected to gas chromatography/time-of-flight (GC-TOF) mass spectrometry–based determinations in order to estimate the concentrations and to adjust the sample dilution prior to the IRMS determinations.

### GC-TOF measurements

All samples were measured on a 7200 Accurate-Mass Quadrupole Time-of-Flight mass spectrometer coupled to a 7890A gas chromatograph (both Agilent, Santa Clara, CA, USA) equipped with J&W Scientific DB-17MS GC column (length 30 m, i.d. 0.25 mm, film thickness 0.25 μm; Agilent). Injections with a volume of 1 μL were performed in pulsed splitless (for vials containing DHEA, EpiA, and A) and in split (1/10) (for vials containing CHOL) mode at 280 °C. He (4.6) was used as the carrier gas at a constant flow of 1.2 mL. The temperature gradient started at 100 °C, held for 2 min, then increased with 40 °C/min to 260 °C, then with 3 °C/min to 289 °C, and then again with 40 °C/min to 320 °C and held for 5 min resulting in a total run time of 21.5 min. The TOF was operated in full scan mode acquiring data at 200 ms/spectrum with an acquisition range from *m*/*z* 50 to 500. Data was evaluated using MassHunter software (version B.06, Agilent). The instrument was mass calibrated using perfluorotributylamine and the software-based algorithm (MassHunter) prior to each sequence in order to achieve mass accuracy in the range of ±5 ppm.

Samples were not quantified but evaluated against a negative quality control serum sample processed with each batch of samples by adopting the volume for reconstitution by a simple rule of proportion. All samples were uncapped and evaporated to dryness in a desiccator before reconstitution with an appropriate volume of cyclohexane and subsequent IRMS determinations.

### Multi-dimensional gas chromatography-combustion-isotope ratio mass spectrometry (MDGC-C-IRMS)

The suitability of MDGC-based approaches to circumvent the time-consuming high-performance liquid chromatography (HPLC)–based sample clean-up step in the context of doping control analysis of endogenous steroids has already been demonstrated in the past [17–19]. Here, the same setup as described by Putz et al. was used within this study and only minor changes deemed necessary [19].

The MDGC was built by connecting two independent Trace GC 1310 gas chromatographs via a pressure-controlled Dean Switch device and a transfer line operated at 300 °C placed in-between both GCs (Thermo, Bremen, Germany). The first GC was additionally equipped with a flame ionization detector (FID, Thermo) operated at 350 °C with a flow rate of 350 mL/min air and 35 mL/min hydrogen in order to estimate appropriate time intervals for sample transfer from the first to the second dimension. The GC column used in the first dimension was an Optima 1 (30-m length, 0.25-mm ID, 1-μm film thickness) from Macherey-Nagel (Düren, Germany). In the second dimension, a J&W Scientific DB-17MS GC column (length 30 m, i.d. 0.25 mm, film thickness 0.25 μm) from Agilent was installed. The effluent of the second GC column was divided between the IRMS and an ISQ single quadrupole mass spectrometer (Thermo). The IRMS was hyphenated to the GC employing a GC IsoLink CNH operated at 950 °C and a ConFlo IV interface (both from Thermo). Injections were performed in splitless mode at 300 °C with 2 to 4 μL cyclohexane using the TriPlus RSH Autosampler (Thermo). Employing the internal standard double mode allowed to co-inject each sample with 1 μL of the internal standard MeT dissolved at 40 μg/mL in cyclohexane.

The GC temperature program in the first dimension depended on the injected analyte. For CHOL, the initial temperature of 100 °C was held for 1.5 min, and then the temperature was raised at 40 °C/min to 330 °C and held constant for 45 min. Typical transfer times between the first and second dimension were 17.3 to 18.3 min for MeT and 28.3 to 29.5 for CHOL. For all other analytes, the initial temperature was at 100 °C for 1.5 min, then with 30 °C/min to 260 °C, and then with 10 °C/min to 300 °C and held for 37 min. Typical transfer times were from 23.3 to 24.2 min for A, 24.3 to 25.1 min for DHEA, 24.8 to 25.8 min for EpiA, and 25.6 to 26.6 for MeT. Overlapping transfer windows were combined as applicable; i.e., each sample containing DHEA, A, and EpiA was injected two times either for measuring EpiA and A or for DHEA, and both injections were accompanied by MeT.

**Fig. 1:**
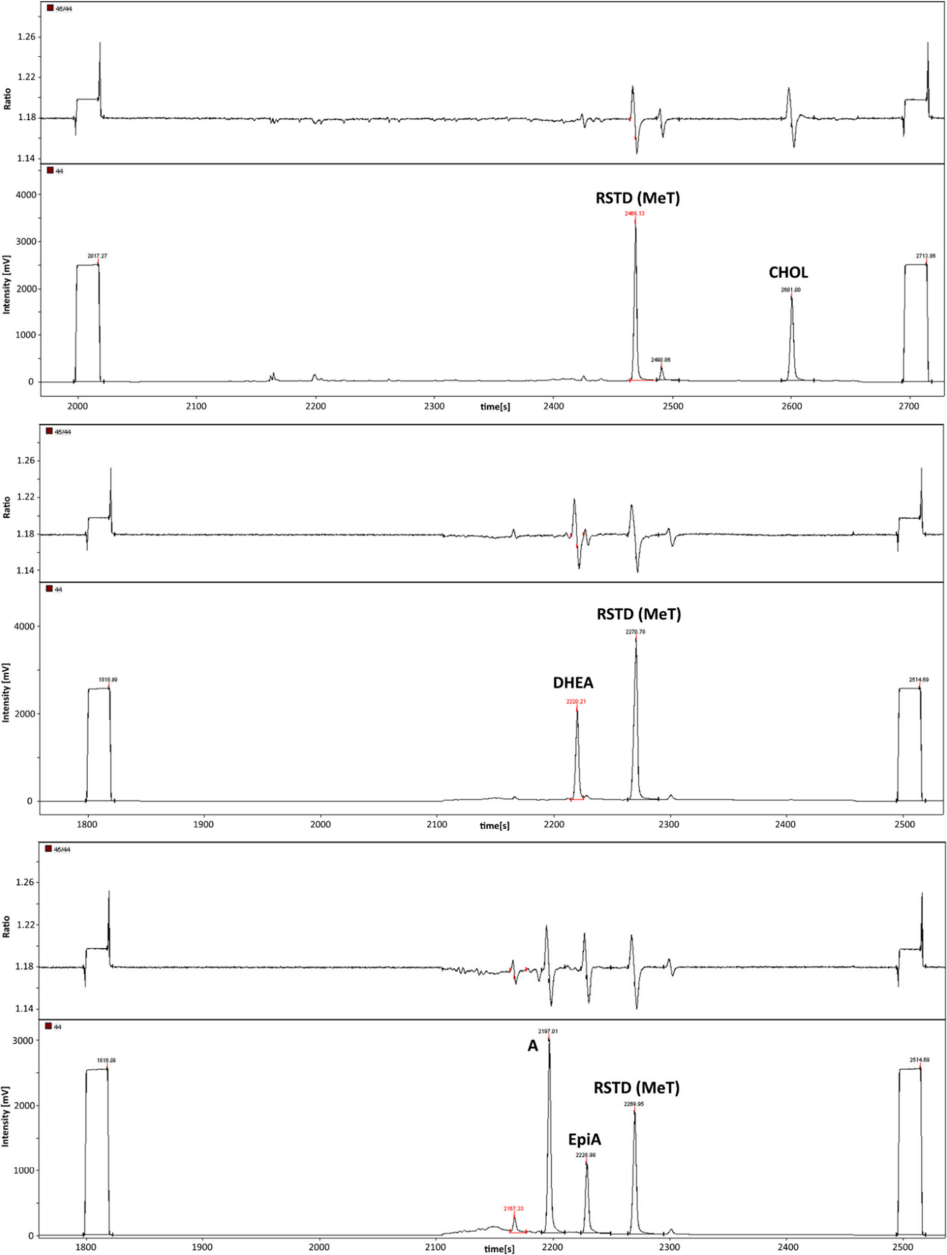
MDGC-C-IRMS chromatograms for all analytes of interest obtained for a quality control serum sample after sample preparation. Upper part: CHOL, middle part: DHEA (both serve as endogenous reference compounds), and lower part: A and EpiA (target analytes). The ratio is given as *m*/*z* 45 divided by *m*/*z* 44

The temperature program in the second dimension was also adopted as needed. For CHOL, the temperature was held at 100 °C for 32 min, then raised at 40 °C/min to 273 °C, and then with 5 °C/min to 320 °C and held for 5 min. The other program remained at 100 °C until 26 min, was then raised at 40 °C/min to 273 °C, then with 2 °C/min to 299 °C, and finally again with 40 °C/min to 320 °C held for 1 min. Typical IRMS chromatograms obtained with this setup are depicted in Fig. 1.

### Method validation

The method was validated on the basis of regulations issued by WADA established for urinary steroids comprising the linear range of the instrument, inter-day and intra-day repeatability, estimation of bias, and the limit of detection (LOD) together with investigations in a reference population [5]. Peak purity, a prerequisite for valid IRMS determinations, was ensured by the described hyphenation of the ISQ to the second GC. During method development, all chromatograms were checked for co-elutions and in all subsequent measurements, the corresponding mass spectra were consulted if the IRMS chromatogram showed signs of possible co-elutions.

#### Linear range of the instrument

The linear range of the MDGC-C-IRMS instrument has been tested by threefold injections of in-house reference material covering a range from 8 to 400 ng of acetylated steroid on column. The different amounts investigated were 8, 20, 32, 40, 100, and 400 ng injected.

#### Repeatability

The intra-day repeatability was tested by sixfold preparation and measurement of a serum sample provided by one volunteer, and the inter-day repeatability was determined by sixfold preparation of a quality control serum sample obtained by pooling presumed negative serum samples from 30 different individuals referred to as negative quality control (NQC) in the following prepared over a time interval of 12 weeks during the course of reference population determinations.

#### Bias

The absence of isotopic fractionation was verified by application of linear mixing models (LMMs) [16, 21, 22]. As no validated method was available in the Cologne doping control laboratory for the determination of steroid concentrations in serum, the concentrations of all analytes of interest were estimated in the NQC by the standard addition technique. The NQC was fortified with increasing amounts of the analytes of interest (employing unconjugated steroids) with maximum concentrations of 500 ng/mL EpiA, 1000 ng/mL A, 4000 ng/mL DHEA, and 50,000 ng/mL CHOL taking into consideration the expected endogenous concentrations of the different steroids.

The LMMs were prepared on two different days by two different analysts fortifying five NQC samples with increasing amounts of unconjugated steroid standards and leaving one blank. The complete LMM approach has been described in detail several times [16, 21, 22]. Results were evaluated by applying a linear regression using “R” version 3.4.3 [23].

#### Limit of detection

As no validated method for the determination of the endogenous steroids in serum under investigation here was available in our laboratory, the results obtained on NQC were used as a basis to estimate the LOD of the developed method.

#### Reference population and calculation of preliminary thresholds

The investigated reference population encompassed 65 athlete serum samples (32 females and 33 males) collected between 2016 and 2018 and stored at −20 °C until analysis. These doping control samples were collected in the context of human growth hormone analysis; all samples were reported negative. Due to the strict demands for anonymous doping control analysis, no information was available regarding a possible misuse of T or T prohormones or nutritional supplements by any of the athletes under investigation. As the results obtained on this population did not comprise any outliers (Grubb’s test, α < 0.01, “R”), the assumption of real negative samples seemed to be justified. Additionally, the population was tested for Gaussian distribution (Shapiro-Wilk test, α < 0.05, “R”) and preliminary thresholds were calculated by adding the threefold standard deviation to the found mean values. Differences between the genders were investigated using the Wilcoxon rank sum test (a < 0.05, “R”).

### Testosterone replacement therapy samples

Serum samples collected in the context of T replacement therapies (intramuscular testosterone undecanoate) in order to determine the serum T concentrations were additionally analyzed with the developed method to evaluate the CIR and test the calculated thresholds for their suitability. One volunteer (VA) provided serum samples directly before starting with the replacement therapy and two times during the application and five volunteers (VB, VC, VD, VE, and VF) provided samples during the replacement therapy at the end of a 12-week interval. All volunteers gave written consent to the additional analysis on CIR.

### Samples derived from a testosterone gel administration trial

One male volunteer (44 years of age, 180-cm height, 82-kg body weight) applied T-Gel on five consecutive days in the evening on arms and breast (two bags of Testogel® containing 50 mg T each, δ^13^C_VPDB_ = −27.3 ± 0.3‰). Both serum and urine samples were collected over a period of 7 days as specified in Table 1. After centrifugation, serum was transferred to Falcon tubes and stored at −20 °C until analysis. Urine samples were prepared according to established protocols and also analyzed employing the MDGC-C-IRMS setup circumventing the HPLC clean-up step [19, 24]. The volunteer gave written consent and the study was approved by the local ethics committee of the German Sport University Cologne (ethical_approval_130404).

**Table 1:** Time points for the collection of serum and urine samples during the T-Gel administration trial

Serum sample	Urine sample	Hours after first application	Hours after last application
S1	U01	−15	−111
S2	U02	9	−87
S3	U03	33	−63
	U04	57	−39
	U05	81	−15
S4	U06	105	9
	U07	114	18
	U08	120	25
S5	U09	129	33
	U10	145	49
S6	U11	153	57

## Results and discussion

### Method validation

All results obtained during method validation demonstrated that the developed method is fit-for-purpose and can be employed in sports drug testing as soon as relevant accreditation bodies have evaluated this novel approach.

#### Linear range of the instrument

**Fig. 2:**
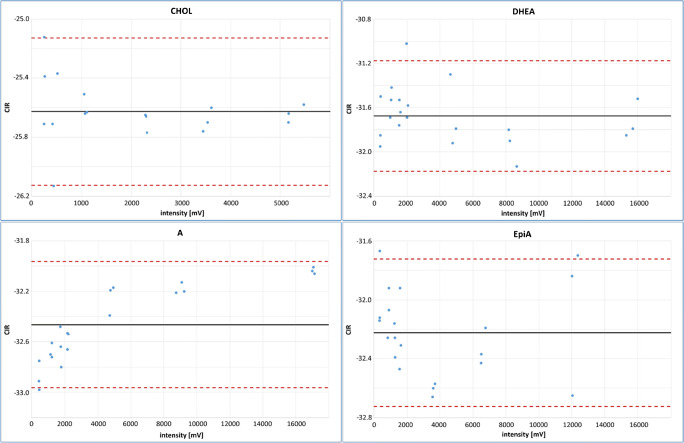
Results obtained for the linearity test of the MDGC-C-IRMS instrument for all analytes of interest. All CIR values in δ^13^C_VPDB_ [‰]. Further information in the text

In IRMS, CIR may show a dependency on the signal intensity and therefore the range of intensities (given in signal heights as mV) providing acceptable results has to be investigated for each analyte of interest. In order to probe for linearity, different amounts of steroids usually provided in nanogram of steroid injected on column are investigated. Comparable linear ranges have been found for all steroids under investigation (CHOL 250–5500 mV for *m*/*z* 44, DHEA 400–15,700 mV, EpiA 350–12,000 mV, and A 440–17,000 mV). CHOL showed a lower response on the MDGC-C-IRMS in general and the lowest amount injected (8 ng on column) did not yield valid results. Therefore, the linear range for CHOL was from 20 to 400 ng on column and from 8 to 400 ng on column for all other analytes as shown in Fig. 2. Only for A, a signal-dependent drift in CIR was noted but remained within the acceptable range of ±0.5‰ as stipulated by WADA [5].

#### Repeatability

The results obtained on repeatability are listed in Table 2. For both the short- and the long-term repeatability, standard deviations (SDs) calculated for the sixfold preparation and measurement of serum samples were found <0.5‰ and therefore in line with results obtained for urinary steroids and even slightly better [22, 25]. In general, the SD of the long-term repeatability was found to be higher to a minor extent when compared to short-term values, which is also in line with doping control methods developed for urinary steroids [14, 16].

**Table 2:** Results obtained on method repeatability. The serum sample analyzed for intra-day results was different from the pooled NQC investigated for inter-day repeatability. All values given in δ^13^C_VPDB_ [‰]

Sample	Intra-day				Inter-day			
	CHOL	DHEA	A	EpiA	CHOL	DHEA	A	EpiA
1	−21.5	−20.7	−21.5	−23.5	−20.5	−20.9	−21.3	−22.5
2	−21.2	−20.9	−21.5	−23.5	−20.7	−20.6	−20.9	−22.3
3	−21.3	−21.1	−21.7	−22.7	−20.3	−20.4	−21.0	−21.9
4	−21.3	−20.6	−21.4	−22.7	−20.5	−20.3	−21.0	−21.5
5	−21.4	−21.0	−22.0	−23.1	−20.7	−21.0	−21.1	−21.4
6	−21.3	−20.7	−21.8	−23.3	−20.4	−21.0	−21.4	−21.5
Mean	−21.3	−20.9	−21.6	−23.2	−20.5	−20.7	−21.1	−21.8
SD	0.10	0.17	0.20	0.32	0.15	0.30	0.18	0.42

#### Bias

No significant isotopic fractionation was found for all steroids under investigation. The 95% confidence interval of the intercept of the linear regression model with the y-axis included the CIR of the standard added for each model. The measurement uncertainty (Mu) was calculated by error propagation according to the following:

3 $$ {M}_u=\sqrt{SE{(a)}^2+ SE{(b)}^2} $$

where *SE* stands for the standard error of the linear regression, *a* for the slope, and *b* for the intercept with the y-axis of the line of best fit [16, 21].

All results are listed in Table 3. A straightforward explanation for the elevated Mu found for A is not available as during the investigations on the reference population, the standard deviations for A were even lower than those for EpiA, and also in the NQC, no discrepancy was noted.

**Table 3:** Results obtained for the linear mixing models together with the calculated measurement uncertainty for the complete method (M_u_). The slope of the line of best fit is represented by *a*, the intercept with the y-axis by *b*, and for both the standard error (SE) is given

Steroid	*a*	SE(*a*)	*b*	SE(*b*)	M_u_
CHOL	3.13	0.16	−26.05	0.10	0.19
DHEA	7.73	0.47	−31.55	0.32	0.57
A	7.81	0.70	−32.48	0.57	0.90
EpiA	5.36	0.57	−31.79	0.43	0.71

##### Limit of detection

Here, only an estimation can be provided based on the concentrations established in the NQC which were found to be at 78 ng/mL for A, 41 ng/mL for EpiA, 336 ng/mL for DHEA, and 46 μg/mL for CHOL. Starting with a volume of 1 mL of serum 30 ng/mL of DHEA, EpiA and A result in valid IRMS signals; for CHOL, no LOD needs to be estimated as here valid measurements are even possible from dried blood spots containing 20 μL of whole blood (unpublished results). Taking into account the results obtained for the reference population, this LOD is fit-for-purpose if 1 mL of serum is available.

##### Reference population and calculation of preliminary thresholds

All measurements conducted in the course of the study on the 65 different individuals yielded valid results within the linear range of the instrument. Only 3 determinations on EpiA were found slightly below the lower end of the linear range but still considered valid as the CIR did not show exceptional values.

**Fig. 3:**
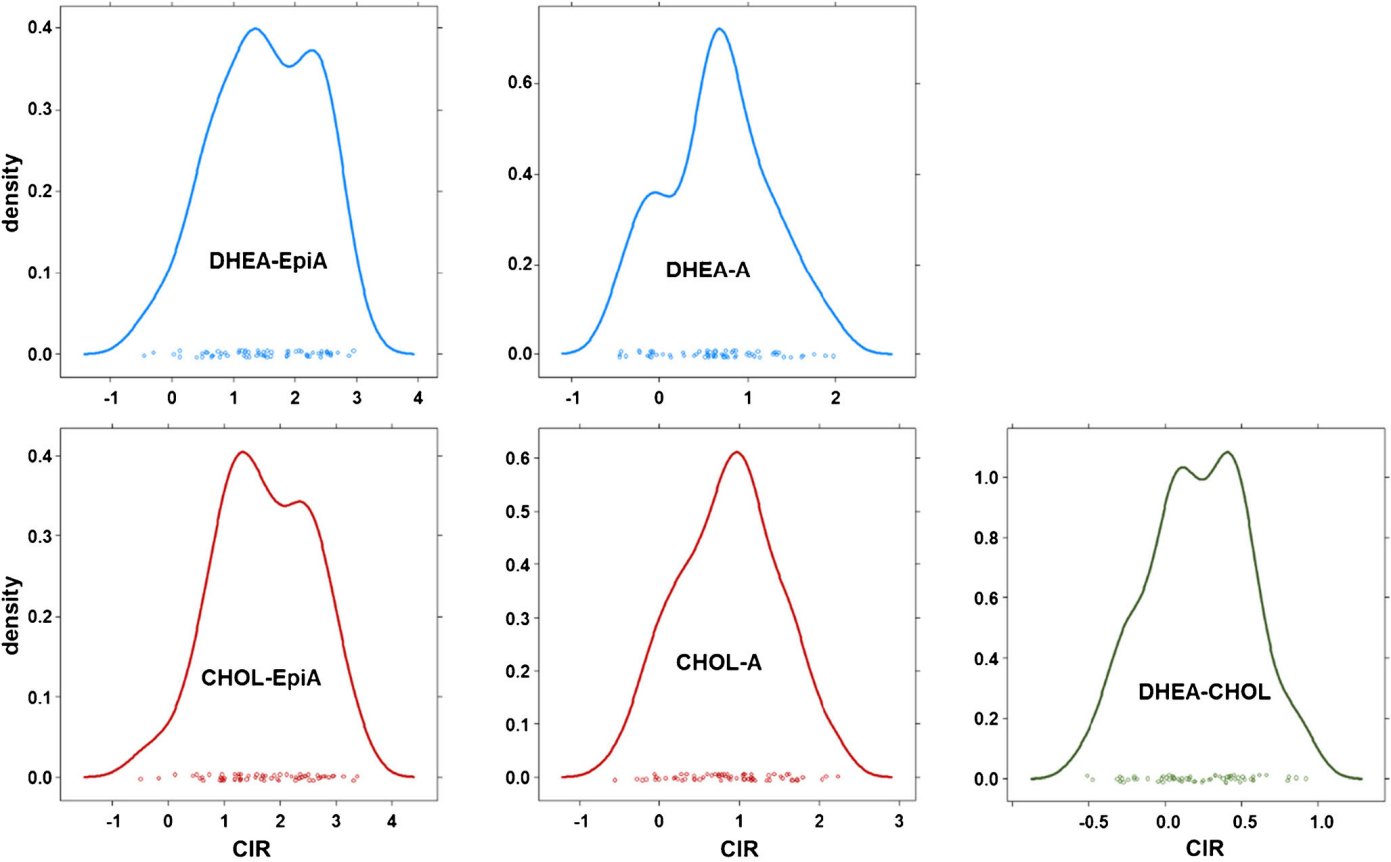
Density plots for all Δ-values under investigation determined in the reference population encompassing n = 65 males and females

**Fig. 4:**
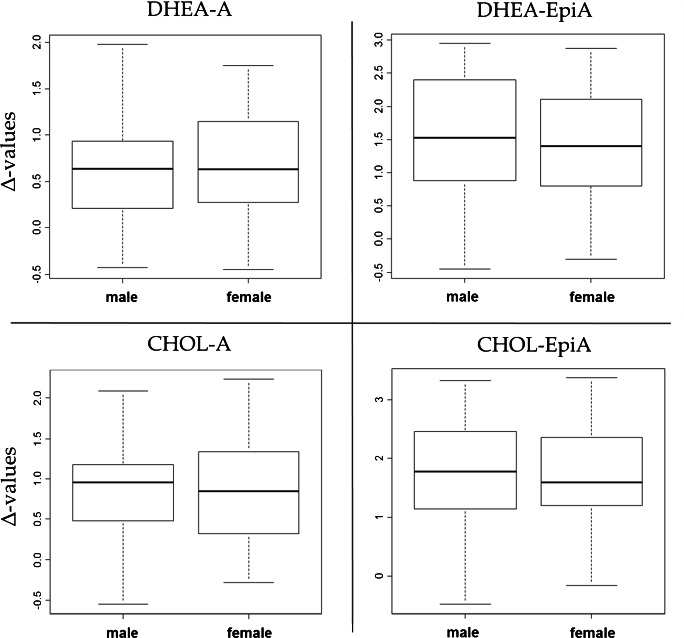
Box plots of all pairs of ERC-TC separated between female and male samples demonstrating no significant difference between the genders

The distribution of δ-values scattered over a large range from −16.7 to −24.8‰ and was therefore comparable to δ-values of steroids found in human urine [25–27]. The calculated Δ-values however were found Gaussian-distributed with a slight tendency to bimodal distributions as depicted in Fig. 3. Density plots, i.e., plots of smoothed histograms, have been chosen here as they allow for a direct visualization of the data’s distribution. Driven by this tendency, a comparison between female and male samples was conducted but neither δ-values nor Δ-values showed any significant difference between the genders (Fig. 4). Increasing the number of participants within the reference population will most probably result in a more uniform distribution of Δ-values. Nevertheless, the results obtained so far allowed to calculate preliminary reference limits based on considerations for clinical testing [28]. The results obtained are listed in Table 4. Comparatively higher thresholds were found for Δ-values including EpiA because of slightly larger SDs and significantly higher mean values due to depleted δ-values found for EpiA compared to A. This is in accordance with values reported for EpiA and A excreted sulfated into urine and will not affect the sensitivity of the method for EpiA as long as the T administered is still significantly depleted compared to the endogenous EpiA δ-values [22, 25]. The reference population–based thresholds were tested by investigating samples derived from T administration trials.

**Table 4:** Reference limits calculated on the basis of n = 65 female and male samples by adding the threefold SD to the mean. All limits were brought up to a round figure. All values given in δ^13^C_VPDB_

	DHEA-A	DHEA-EpiA	CHOL-A	CHOL-EpiA	DHEA-CHOL
Mean	0.54	1.49	0.82	1.77	0.30
SD	0.56	0.83	0.59	0.88	0.32
Limit	2.3	4.0	2.6	4.5	1.3

### Testosterone replacement therapy samples

Overall, serum samples from 6 different male volunteers were investigated (Table 5). Volunteer VA agreed to participate prior to the start of the replacement therapy. So, in this case, a pre-administration sample was available. The baseline sample (VA-T0) fits perfectly into the distribution obtained for the reference population. Both samples collected after the therapy started (VA-T1 and VA-T2) showed significantly depleted CIR for both TCs and the resulting Δ-values clearly indicate the exogenous source of A and EpiA. The other sample donors (VB to VF) suffered from male hypogonadism received 500 to 1000 mg testosterone undecanoate (Nebido®, Jenapharm) as part of their usual treatment regimen 10 to 12 weeks before serum sample collection (trough levels). All serum samples fall beyond the established population-derived thresholds for Δ-values. Only in volunteer E, the Δ-values calculated with CHOL as ERC were already unsuspicious, while using DHEA as ERC could still prove the administration. This finding can either be attributed to a lower administered dose (500 mg T undecanoate) or to a larger endogenous dilution caused by a higher endogenous production of target steroids in the body or both. As the administration of a large dose of a T-ester seems rather unlikely in the course of a doping scenario, the impact of T-Gel administration was additionally investigated.

**Table 5:** CIR obtained in serum of male volunteers participating in T replacement therapies. All values given in δ^13^C_VPDB_. Further information in the text

Volunteer	A	EpiA	DHEA	CHOL	DHEA-A	DHEA-EpiA	CHOL-A	CHOL-EpiA
VA-T0	−23.9	−24.6	−22.8	−22.8	1.1	1.7	1.1	1.7
VA-T1	−27.5	−27.8	−22.9	−23.0	***4.6***	***4.9***	***4.5***	***4.8***
VA-T2	−27.0	−28.2	−23.0	−22.7	***4.0***	***5.2***	***4.2***	***5.4***
VB	−30.0	−31.1	−23.1	−23.9	***6.8***	***8.0***	***6.0***	***7.2***
VC	−26.2	−27.7	−21.4	−21.1	***4.8***	***6.2***	***5.1***	***6.6***
VD	−29.7	−31.2	−23.0	−23.6	***6.7***	***8.2***	***6.0***	***7.6***
VE	−24.0	−24.7	−20.6	−21.8	***3.3***	***4.0***	2.2	2.9
VF	−29.5	−28.5	−24.3	−24.0	***5.2***	***4.2***	***5.4***	***4.4***

Numbers in bold/italic represent values found above relevant thresholds demonstrating the exogenous origin of TCs

### Samples derived from a testosterone gel administration trial

**Fig. 5:**
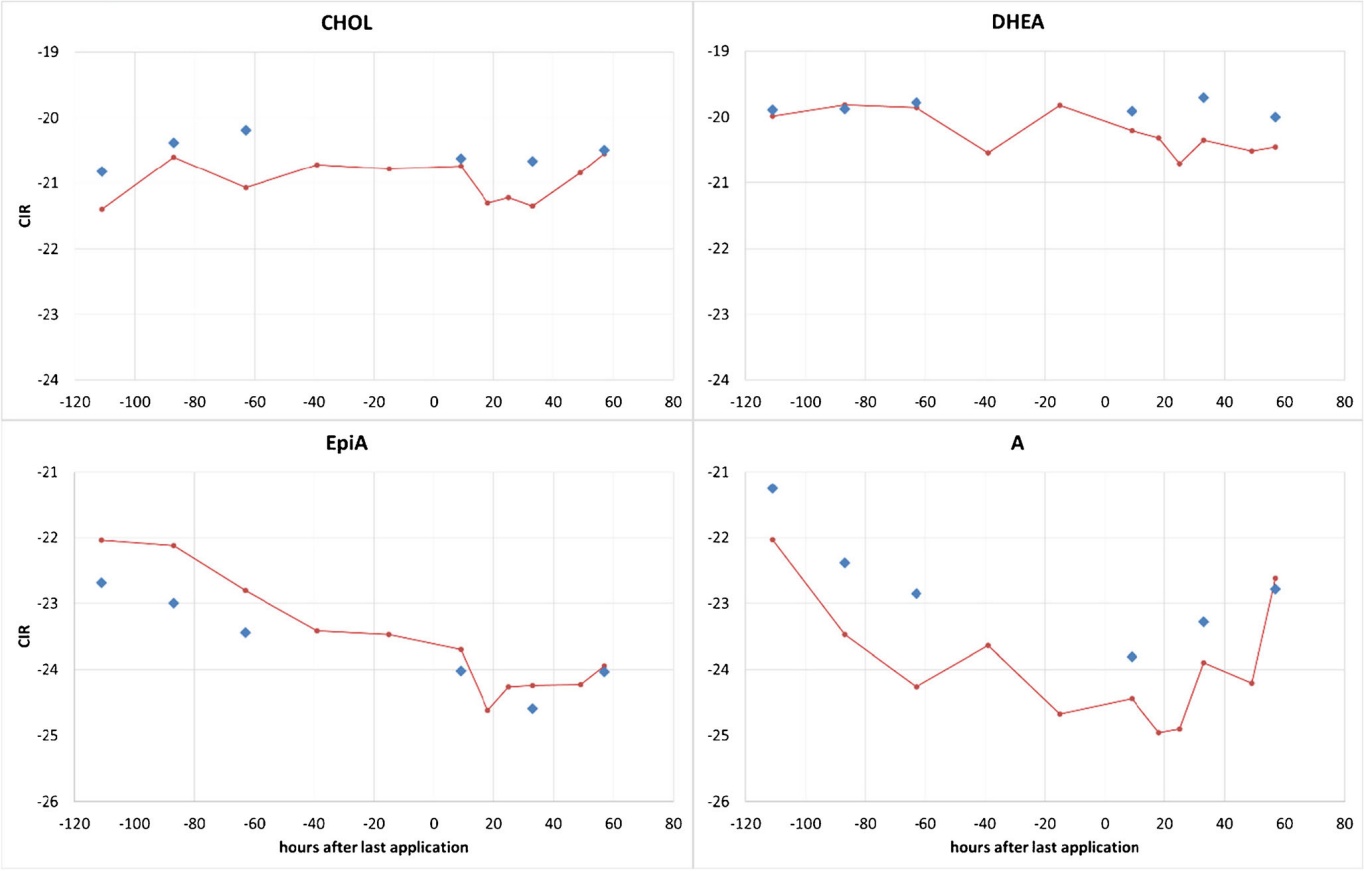
CIR obtain after the fivefold administration of 100 mg T-Gel per day to one male volunteer. The red line represents the CIR found in urine, the blue diamonds the values found in serum. In both biological matrices, the sulfo-conjugated steroids were investigated. All values given in δ^13^C_VPDB_

During the administration trial, urine and serum samples were collected in parallel at six different time points as listed in Table 1, allowing for a direct comparison of steroids found in different matrices. For all 4 steroids under investigation, a parallel trend in their CIR for urine and serum was noted as shown in Fig. 5. Both ERCs (DHEA and CHOL) remained stable over the complete administration trial with SDs < 0.3‰. The TCs showed a constant depletion in their CIR over the course of the study with a faster and more pronounced impact on A and a slower but prolonged impact on EpiA. The slower turnover of EpiA has already been described for this steroid after being excreted into urine [22, 24]. Due to the relatively large endogenous pool size of both sulfo-conjugated A and EpiA and the resulting endogenous dilution, both do not reach the CIR of the administered T (−27.3‰). Nevertheless, the impact on Δ-values was found strong enough to fall beyond the population-derived thresholds as demonstrated in Table 6.

**Table 6:** CIR obtained in a male volunteer after fivefold application of 100 mg T-Gel. Steroids measured in serum were sulfo- or unconjugated, steroids from urine were glucuro-conjugated. Numbers in bold/italic represent values found above relevant thresholds demonstrating the exogenous origin of TCs. All values given in δ^13^C_VPDB_

Hours after last administration	Serum				Urine			
	DHEA-A	DHEA-EpiA	CHOL-A	CHOL-EpiA	PD-A	PD-ETIO	PD-5a	PD-5b
−111	1.4	2.8	0.4	1.9	0.2	0.3	0.1	0.7
−87	***2.5***	3.1	2.0	2.6	***2.3***	1.7	***3.3***	2.9
−63	***3.1***	3.7	***2.7***	3.3	***3.1***	2.3	***3.6***	2.8
9	***3.9***	***4.1***	***3.2***	3.4	***4.0***	2.9	***5.2***	2.8
33	***3.6***	***4.9***	***2.6***	3.9	***2.4***	1.6	***3.7***	2.6
57	***2.8***	***4.0***	2.3	3.5	0.6	0.8	1.0	1.7

Especially for A and using DHEA as ERC, depleted CIR values are visible from the beginning until 57 h after the last administration. Using CHOL, values demonstrated the exogenous origin of the TC for 33 h. EpiA also exhibits depleted ^13^C values from the first administration on, but only exceeds the limit of respective Δ-values after cessation of administration and then until the end of the study when employing DHEA as ERC. Utilizing CHOL, the impact on Δ-values was also noted but did not reach the population-based thresholds. For comparison, the values found in urine for those glucurono-conjugated steroids investigated in routine doping controls are additionally listed in Table 6. Here, the well-known pronounced impact on steroids with a 5α configuration (A and 5a) is clearly visible [21]. The steroids with 5β configuration (ETIO and 5b) also showed depleted CIR but did not exceed the relevant thresholds [5]. Urinary steroids do only show significantly depleted values until 33 h after the last application. The prolonged detection window in serum is most probably due to the employment of sulfo-conjugated steroids in this biological matrix. Further studies involving different volunteers seem to be necessary to evaluate the sensitivity of serum steroids comprehensively.

## Conclusion

A method to determine the CIR of endogenous steroids found in human serum has been developed and validated in accordance with current WADA regulations for urinary steroids. Employing an MDGC-C-IRMS-based approach allowed to circumvent HPLC sample clean-up and to employ serum volumes for sample preparation ≤1 mL with an estimated concentration of 30 ng/mL as LOD. The investigated reference population enabled to calculate preliminary thresholds that can straightforwardly be applied to distinguish between naturally occurring CIR and depleted CIR caused by the administration of an exogenous steroid. Applying these thresholds to samples derived from T replacement therapies clearly demonstrated their applicability. As the doses administered during replacement are unrealistically high with regard to doping scenarios, a T administration trial encompassing a 5-day application of T-Gel was additionally investigated. The serum steroids showed significantly depleted CIR and resulted in slightly longer detection windows compared to urinary steroids. Summarizing these promising results, the developed method is considered to be fit-for-purpose. Nevertheless, additional experiments seem to be expedient in order to further investigate the sensitivity of the new method towards low-dose T administrations. Expanding the size of the reference population may further increase statistical confidence in the established thresholds.

## Declarations

### Source of biological material

Samples investigated as reference population were collected doping control samples anonymized before use, excretion study samples have been collected at the German Sport University Cologne and patient samples were collected at the University Hospital of Muenster and the University of Bern.
